# Short- and midterm outcome of ruptured and unruptured intracerebral wide-necked aneurysms with microsurgical treatment

**DOI:** 10.1038/s41598-021-84339-x

**Published:** 2021-03-02

**Authors:** Sae-Yeon Won, Volker Seifert, Daniel Dubinski, Sepide Kashefiolasl, Nazife Dinc, Markus Bruder, Juergen Konczalla

**Affiliations:** Department of Neurosurgery, University Hospital, Goethe-University, Frankfurt am Main, Germany

**Keywords:** Neurology, Outcomes research

## Abstract

To clip or coil has been matter of debates for several years and is the domain of interdisciplinary decision making. However, the microsurgical outcome has still been elusive concerning wide neck aneurysms (WNA). A retrospective single center study was performed with all patients with ruptured WNA (rWNA) and unruptured WNA (uWNA) admitted to author´s institute between 2007–2017. Microsurgical outcome was evaluated according to Raymond-Roy occlusion grade and follow-up angiography was performed to analyze the stability of neck/aneurysm remnants and retreatment poverty. Of 805 aneurysms, 139 were rWNA (17.3%) and 148 uWNA (18.4%). Complete occlusion was achieved in 102 of 139 rWNA (73.4%) and 112 of 148 uWNA (75.6%). Neck remnants were observed in 36 patients with rWNA (25.9%) and 30 patients with uWNA (20.3%), 1 (0.7%) and 6 (4.1%) patients had aneurysmal remnant, respectively. Overall complication rate was 11.5%. At follow-up (939/1504 months), all remnants were stable except for one, which was further conservatively treated with marginal retreatment rate under 1%. Even the risk of de-novo aneurysm was higher than the risk for remnant growth (2.6% vs 0% in rWNA; 8.7% vs 5.3% in uWNA) without significant difference. Microsurgical clipping is effective for complete occlusion of r/uWNA with low complication. Furthermore, the risk of remnant growth is marginal even lower than the risk of de-novo rate low retreatment rate.

## Introduction

The question between clip and coil has been matter of debates for the past decades. Since International Subarachnoid Aneurysm trial (ISAT), one could divide the medical care of ruptured aneurysm into pre- and post-era ISAT with a dramatic increase of endovascular treatment^1,2^. Similar results were reported in Barrow Ruptured Aneurysm Trial in the first year of follow-up, but at further follow up (3-/6- and recently 10 years), there was no difference in terms of patient´s outcome between those two modalities (especially in anterior circulation aneurysms) except higher recanalization and retreatment rate in the endovascular group resulting in an increased valuability of surgical treatment^3,4^ Hence, both treatment modalities have its advantages and disadvantages and the decision for aneurysmal treatment is made by interdisciplinary consensus. In particular, the focus of those trials rely on saccular aneurysms; however, microsurgical outcome regarding wide neck aneurysms (WNA), as known as complex form of aneurysm, is still elusive.

Recently, Mascitelli et al. reported significant better microsurgical outcome of ruptured WNA compared to endovascular treatment in the subgroup analysis of Barrow Ruptured Aneurysm Trial^11^. In contrast to the several studies reporting diverse novel technique for the endovascular treatment of WNA, there is paucity of studies reporting surgery in WNA leading to the insufficient database to compare those modalities appropriately. Therefore, we sought to analyze and complement the microsurgical outcome of ruptured and unruptured WNA in order to answer the following questions: (1) How many ruptured and unruptured WNA are completely or adequately occluded? (2) Are the microsurgical treated WNAs stable at follow up or how are the rates of remnant growth compared to de-novo rate? (3) What are the factors for postoperative remnant?

## Results

### Basic characteristics, clinical course and functional outcome

Of 805 patients in our database between 2007 and 2017, 287 patients had a WNA (35.7%). In this series, a total of 139 patients (48.4%) had a ruptured WNA and 148 patients (51.6%) had an unruptured WNA.

Regarding patients with rWNA, the median age was 52 years of age (IQR 20–83) and 93 of 139 patients (66.9%) were female. About half of those patients were in good admission status (WFNS 1–3; 50%). Early hydrocephalus occurred in 95 patients (69.1%) and cerebral vasospasm (CVS) manifested in 106 of 139 patients (76.3%) with DCI in over 50% (73 of 139 patients). Favorable outcome (mRS 0–2) was achieved 37 patients at discharge (25.9%) and in 75 patients at FU (56%). Detailed information is listed in Table 1.

**Table 1:** SAH-Patient characteristics and outcome at discharge as well as at follow up.

Variable	Value
No. of patients	139
No. of patients with multiple aneurysms	43
Median age, yrs (range)	52 (20–83)
**Sex**	
Male	46 (33.1%)
Female	93 (66.9%)
Smoker	59 (42.1%)
WFNS 4–5 at admission	69 (49.6%)
**WFNS**	
1	42 (30.2%)
2	15 (10.8%)
3	13 (9.4%)
4	10 (7.2%)
5	59 (42.4%)
**Modified CT-Fisher score**	
1	7 (5%)
2	12 (8.6%)
3	73 (52.6%)
4	47 (33.8%)
**ICH**	47 (33.8%)
< 50 ml	30 (21.6%)
> 50 ml	17 (12.2%)
Hydrocephalus	96 (69.1%)
**CVS**	
None	29 (20.9%)
Mild (< 33% luminal narrowing)	23 (16.5%)
Moderate (33–66% luminal narrowing)	41 (29.5%)
Severe (> 66% luminal narrowing)	42 (30.2%)
Undefined	4 (2.9%)
DCI	73 (52.5%)
**Favorable outcome (MRS0-2) at discharge**	36 (25.9%)
Outcome at discharge	139
1	23 (16.5%)
2	13 (9.4%)
3	14 (10.1%)
4	31 (22.3%)
5	44 (31.6%)
6	14 (10.1%)
**Favorable outcome (MRS0-2) at 6 months**	75 (56%)
Outcome at 6 months	133 (95.7%)
0	20 (15%)
1	33 (24.8%)
2	22 (16.5%)
3	9 (6.8%)
4	22 (16.6%)
5	8 (6%)
6	19 (14.3%)

*SAH* subarachnoid hemorrhage, *yrs* years, *WFNS* World Federation of Neurological Surgeons, *ICH* intracerebral hemorrhage, *CVS* cerebral vasospasm, *DCI* delayed cerebral ischemia, *MRS* modified Rankin scale.

Regarding patients with uWNA, basic characteristics (age, sex distribution) were comparable to rWNA-group. Eighteen of 148 patients (12.2%) had previous SAH. Favorable outcome (mRS 0–2) was achieved in 137 of 148 patients (92.6%) at discharge and 140 of 148 patients (94.6%) at 6 months’ FU. Of note, 4 patients with uWNA, who were admitted in our clinic due to SAH from another aneurysm, were included in the analysis. All of them had initially worse admission status. Further details are reported in Table 2.

**Table 2:** Patient and aneurysm characteristics in unruptured wide-neck aneurysm.

Variable	Value
No. of patients	148
No. of patients with multiple aneurysms	73 (49.3%)
Median age, yrs (range)	52 (16–73%)
**Sex**	
Male	36 (24.3%)
Female	112 (75.7%)
Familiar history of aneurysm	12 (8.1%)
Previous SAH	18 (12.2%)
Smoker	79 (53.4%)
**Favorable outcome (MRS0-2) at discharge**	137 (92.6%)
Outcome at discharge	148
0	71 (48%)
1	54 (36.5%)
2	12 (8.1%)
3	2 (1.3%)
4	7 (4.7%)
5	2 (1.3%)
**Favorable outcome (MRS0-2) at 3–6 months**	140 (94.6%)
Outcome at 3–6 months	148
0	103 (69.6%)
1	29 (19.6%)
2	8 (5.4%)
3	5 (3.4%)
4	3 (2%)
5	0 (0%)

### Aneurysm characteristics, microsurgical treatment and short-term outcome

**Figure 1:**
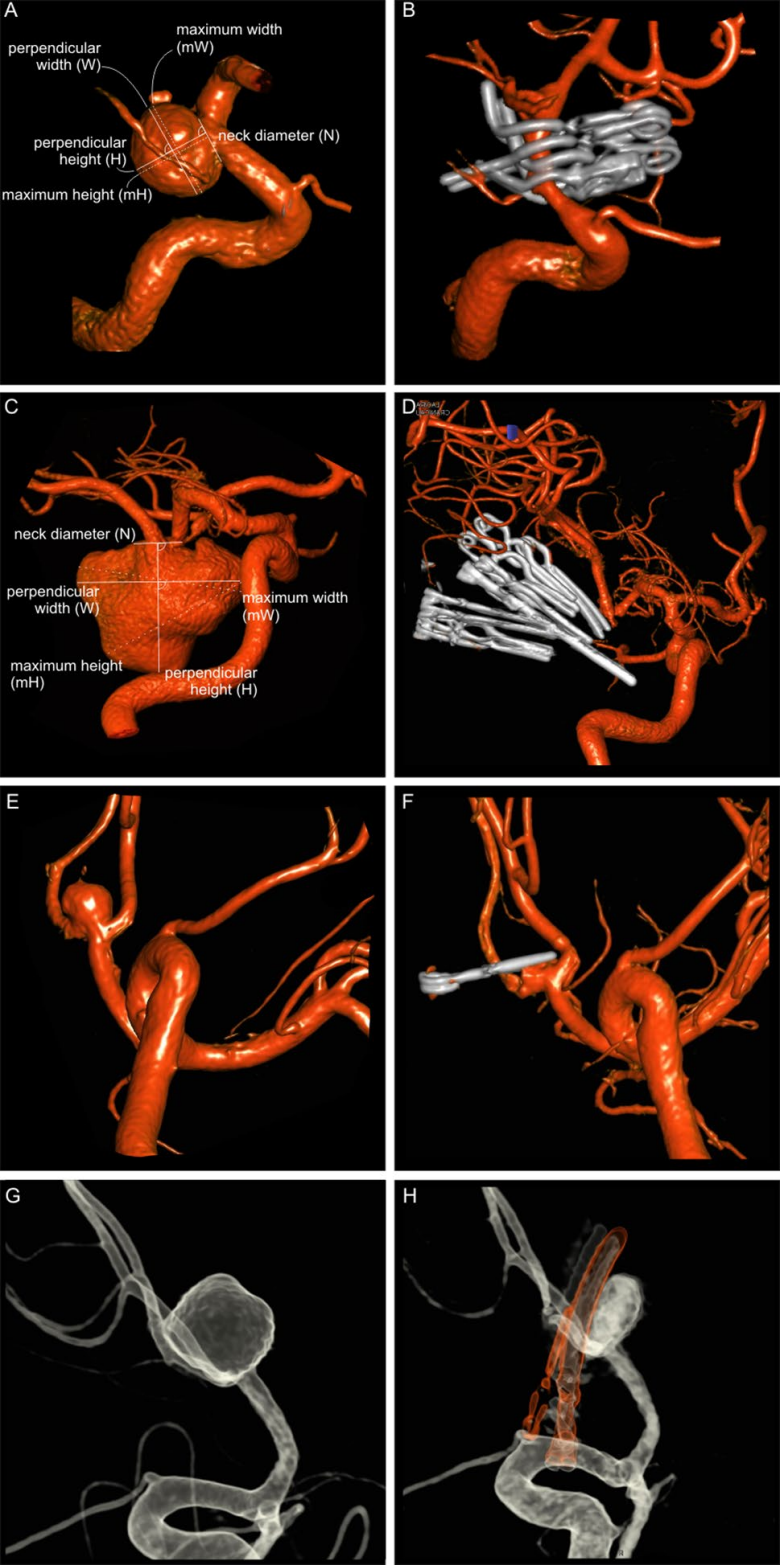
(**A**,**B**) A case with a saccular perpendicular ICA-Pcomm aneurysm and its complete occlusion by 4 clips. (**C**,**D**) A case with a nonsaccular non-perpendicular MCA aneurysm and its complete occlusion by 9 clips. (**E**,**F**) A case with AcommA aneurysm and its neck remnant. (**G**,**H**) A case with AcommA aneurysm and its aneurysmal remnant. Afterwards, postoperative endovascular treatment of the remnant was performed. *ICA* interal carotid artery, *Pcomm* posterior communicating artery, *MCA* media cerebral artery, *AcommA* anterior communicans artery.

The most common rWNA was MCA- (50 of 139; 36%) followed by AcommA- (38 of 139; 28.1%) and ICA- aneurysm (28 of 139; 20.1%), particularly at the location of Pcomm (17 of 28; 60.7%). Mean neck size was 5.0 ± 2.0 mm and over 70% of aneurysm showed dome to neck ratio under 2. 94 of 139 rWNA (67.6%) had saccular perpendicular form of aneurysm and 102 of 139 rWNA (73.4%) were microsurgically completely occluded (Fig. 1A,B). In 36 of 139 patients (25.9%), there was a neck remnant (25.9%) and one patient (0.7%) had a residual aneurysm, which was just conservatively controlled at follow-up. For the aneurysm occlusion, mean number of clips was 1.8 ± 1.2. By doing so, volume reduction of rWNA was postoperatively achieved up to 85%. In total, the complication rate of operation was 15.2%. In 10 cases (7.2%), there were intraoperative rupture of those aneurysms, which were all surgically managed without relevant intraoperative complication except the necessity of blood product transfusion. Of note, relevant complication requiring revision due to secondary epidural hematoma or insufficient clipping was found in 4 of 139 patients (2.9%) and 4 of 139 patients (2.9%) had brain insult (4–13.5 cm^3^) without relevant clinical impairment (Table 3). The brain insult occurred due to Heubner artery occlusion (two patients), perforator vessel injury (one patient) and micro embolism after clip reposition (one patient).

**Table 3:** Characteristic of ruptured aneurysms and microsurgical treatment with follow up.

Variable	Value
No. of patients	139
No. of aneurysms	242
**Location of ruptured aneurysm (n = 139)**	
ACA:	44 (31.7%)
A1	3 (2.2%)
AcomA	38 (28.1%)
A2	2 (1.4%)
A3/4	1 (0.7%)
**ICA:**	28 (20.1%)
Opthalmic	5 (3.6%)
Pcomm	17 (12.2%)
Bifurcation	6 (4.3%)
**MCA**:	60 (43.2%)
M1	3 (2.2%)
MCA-bifurcation	50 (36%)
M2	5 (3.6%)
M3/4	2 (1.4%)
Posterior circulation	7 (5.0%)
PCA	2 (1.4%)
PICA	3 (2.2%)
VA	2 (1.4%)
Aneurysm size, mm ± SD (range, mm)	9.0 ± 4.2 (4–28)
≥ 10 mm	51 (36.7%)
≥ 25 mm	4 (2.9%)
Neck size, mm ± SD	5.0 ± 2.0
Non-Saccular non-perpendicular form of aneurysm (2 mm cutoff)	45 (32.4%)
**Dome to neck ratio**	
< 1	25 (18%)
1–1.9	73 (52.5%)
2–2.9	35 (25.2%)
≥ 3	6 (4.3%)
No. of clips	1.8 ± 1.2
**Aneurysm occlusion grade**	
Complete occlusion	102 (73.4%)
Neck remnant	36 (25.9%)
Residual aneurysm	1 (0.7%)
Aneurysm volume (ml)	190.6 ± 330.3
Postoperative remnant volume (ml)	28.5 ± 221.3
Percentage of volume reduction	85%
Complication of operation	21 (15.1%)
Intraoperative rupture	10 (7.2%)
Secondary epidural hemorrhage without revision	1 (0.7%)
Secondary epidural hemorrhage with revision	2 (1.4%)
Brain insult on CT	4 (2.9%)
Vascular stenosis without revision	2 (1.4%)
Revision with clip replacement	2 (1.4%)
Follow up (n = 51), months	949
Mean ± standard deviation, months	18.6 ± 19.4

*ACA* anterior cerebral artery, *AcomA* anterior communicans antery, *ICA* internal carotid artery, *MCA* media cerebral artery, *PCA* posterior cerebral artery, *PICA* posterior inferior cerebellar artery, *VA* vertebral artery, *CT* computer tomography.

The location distribution of uWNA was similar to rWNA except there was more ophthalmic ACI-aneurysm than Pcomm ICA-aneurysm (10.1% vs 5.4%). Mean neck size was 5.3 ± 2.6 mm and 77.7% showed dome to neck ratio under 2. 71 of 148 uWNA (48%) had saccular perpendicular form of aneurysm and 112 of 148 uWNAs (75.6%) were microsurgically completely occluded (Fig. 1C,D). Neck remnant was observed in 30 of 149 uWNA (20.3%; Fig. 1E,F) and 6 of 148 patients (4.1%) had a residual aneurysm (Fig. 1G,H). One of the residual aneurysm (0.7%) was endovascularly treated at follow-up. For the aneurysm occlusion, mean number of clips was 2.1 ± 1.4. Overall 88% volume reduction of uWNA was postoperatively achieved. In total, complication rate was 8.1%, including intraoperative rupture (2%) and abruption of operation due to brain swelling under seizure (2%) (Table 4). Among six patients (4.1%) with brain insult (3–10.5 cm^3^), it occurred due to perforator vessel injury (four patients) and micro embolism after clip reposition (two patients). Notably, no revision operation was necessary in uWNA treatment.

**Table 4:** Aneurysm characteristic and surgical treatment with follow-up in unruptured wide-neck aneurysm.

Variable	Value
No. of patients	148
No. of aneurysms	241
Location of aneurysm (n = 148) ACA:	34 (23.0%)
A1	3 (2%)
AcommA	25 (16.9%)
A3/4	6 (4.1%)
**ICA:**	28 (18.9%)
Ophthalmisch	15 (10.1%)
Pcomm	8 (5.4%)
Bifurcation	5 (3.4%)
**MCA**:	84 (56.8%)
M1	6 (4.1%)
MCA-bifurcation	73 (49.3%)
M2	5 (3.4%)
Posterior circulation	2 (1.4%)
PICA	1 (0.7%)
VA	1 (0.7%)
Aneurysm size, mm ± SD (range, mm)	9.4 ± 5.2 (4–29)
≥ 10 mm	50 (33.8%)
≥ 20 mm	8 (5.4%)
Neck size, mm ± SD	5.3 ± 2.6
Perpendicular form of aneurysm (1 mm cutoff)	71 (48%)
**Dome to neck ratio**	
< 1	26 (17.6%)
1–1.9	89 (60.1%)
2–2.9	27 (18.2%)
≥ 3	6 (4.1%)
No. of clips	2.1 ± 1.4
**Aneurysm occlusion grade**	
Complete occlusion	112 (75.6%)
Neck remnant	30 (20.3%)
Residual aneurysm	6 (4.1%)
Aneurysm volume	270.1 ± 683.2
Postoperative remnant volume	32.3 ± 300.6
Percentage of volume reduction	88.1%
Complication of operation	12 (8.1%)
Intraoperative rupture	3 (2%)
Brain insult	6 (4.1%)
Abruption of operation due to brain swelling under seizure	3 (2%)
Follow up (n = 69), months	1509
Mean ± standard deviation, months	22.2 ± 19.9

### Microsurgical mid-term outcome

All patients with rWNA and uWNA were clinically followed up; however, radiological follow-up was performed in selected patient with either considerable aneurysm remnant or diagnosed additional aneurysms. 51 of 139 rWNA patients (36.7%) with 949 follow-up months (mean of 18.6 ± 19.4 months) and 68 of 148 uWNA patients (45.9%) with 1509 follow-up months (mean of 22.2 ± 19.9 months) were analyzed. Among 51 patients with rWNA, 38 (74.5%) were initially completely occluded and no recurrent aneurysm was detected at mean follow up of 14.9 ± 14.6 months. There was one de-novo aneurysm (2.6%) in other localization detected. 13 of 51 rWNA (25.5%) had aneurysm remnants, 12 neck- and 1 residual aneurysm. Even in 1 of 12 patients with neck-remnant (8.3%), the remnant disappeared and all other aneurysms were stable without any sign of growth at mean follow up of 29.9 ± 28.2 months. The residual aneurysm was stable up to 23 months’ follow-up (Table 5A). In the uWNA group, 46 of 68 uWNA (67.6%) were initially completely occluded and there was no recurrent aneurysm detected at mean follow up of 24.2 ± 20.9 months. Four de-novo aneurysm (8.7%) were detected in other localization. After initial microsurgical treatment, 19 patients had neck remnants (27.9%) and 3 patients had residual aneurysms (4.4%). 18 of 19 neck remnants were stable up to 19.6 ± 18.9 months and 1 of 19 showed aneurysm growth at follow up of 18 months, however, no additional treatment was required. Among three residual aneurysms, in one case the residual aneurysm disappeared (33%) and the remnant was stable at mean follow up of 7 ± 4.2 months (Table 5B). To illustrate the case, one patient had a media bifurcation aneurysm of a size of 12 mm and was electively clipped (Supplementary Figure e-1A). Three days after operation, an angiography was performed which showed a filiform aneurysmal remnant of a size of 6.5 mm (Supplementary Figure e-1B). It was conservatively treated and one year afterwards, the aneurysmal remnant was not detectable in the angiography indicating spontaneous thrombosis of the remnant (Supplementary Figure e-1C).

**Table 5:** Follow-up analysis after microsurgical treatment.

A) Ruptured wide-neck aneurysms				
n = 51	No remnant	Remnant stable	Remnant growth	De-novo
Complete occlusion (n = 38)	38 (100%)	0 (0%)	0 (0%)	1 (2.6%)
Neck remnant (n = 12)	1 (8.3%)	11 (91.7%)	0 (0%)	0 (0%)
Residual aneurysm (n = 1)	0 (0%)	1 (100%)	0 (0%)	0 (0%)

B) UNRUPTURED wide-neck aneurysms				
n = 68	No remnant	Remnant stable	Remnant growth	De-novo
Complete occlusion (n = 46)	46 (100%)	0 (0%)	0 (0%)	4 (8.7%)
Neck remnant (n = 19)	0 (0%)	18 (94.7%)	1 (5.3%)	0 (0%)
Residual aneurysm (n = 3)	1 (33.3%)	2 (66.7%)	0 (0%)	0 (0%)

### Predictors for microsurgical WNA remnants

In the univariate analysis of rWNA cohorts, predictors for aneurysm remnants were worse admission status of patients (OR 2.7 Cl 95% 1.2–6.1), development of early hydrocephalus (OR 9.1 Cl 95% 3.5–23.8) and non-saccular non-perpendicular form of aneurysm (OR 2.5 Cl 95% 1.0–5.0). Aneurysm of vertebral artery showed a trend towards significance. Interestingly the neck sizes as well as the maximum aneurysm size were not significant predictors (Supplementary Table e-1). In the multivariate analysis of same cohorts, two independent predictors, early hydrocephalus (OR 3.2 Cl 95% 1.2–8.8) and non-saccular non-perpendicular (complex) form of aneurysm (OR 2.0 Cl 95% 1.0–5.0) were identified (Nagelkerke R^2^ = 0.206) (Supplementary Table e-1).

In the univariate analysis of uWNA cohorts, worse admission status of patients, neck size, and non-perpendicular form of aneurysm showed tendency towards significance (Supplementary Table e-2). Due to the fact, multivariate analysis was not possible in this cohort of patients.

## Discussion

### Main findings

There are three main findings in this study. First, the complete occlusion rate of rWNA as well as uWNA is over 70% and stays stable at mid-term follow-up. Even the occurrence rate of de-novo aneurysm is higher compared to the risk of remnant growth indicating absolute stability of microsurgical outcome (Table 6A,B). Second, the procedural complication rate with 11.5% and retreatment rate of less than 1% at follow-up are reasonable values for the preference of surgical treatment of WNA (Table 6C). Third, independent predictors for remnant are neither the size of neck nor aneurysm but early hydrocephalus, complex configuration of aneurysm and number of clips, which were used.

**Table 6:** Summary of microsurgical treatment for wide neck aneurysm.

A. Short-term analysis (at discharge)			
Number of aneurysms	Complete	Neck remnant	Aneurysmal remnant
287	214 (74.6%)	66 (23%)	7 (2.4%)

B. Mid-term analysis (18 to 24 months)			
Number of aneurysms	Complete	Neck remnant	Aneurysmal remnant
119	86 (72.3%)	30 (25.2%)	3 (2.5%)

C. Procedural complication and retreatment at follow-up			
Case no.	Complication	Retreatment	
287	33 (11.5%)	1 (0.3%)	

To clip or coil was and is still matter of debates, however, the direction of aneurysmal treatment dramatically changed since the publication of ISAT (International Subarachnoid Aneurysm Trial), which indicated better clinical outcome of endovascular arm at 1 year- up to 18 years of follow-up if treatment associated deaths within the first year were excluded from analysis^1,2^. On the other hand, ISAT itself (5-year data) and BRAT (Barrow Ruptured Aneurysm Trial) trial reported no significant difference of outcome at 3-, 6- and 10-years follow-up despite the higher likelihood of poor outcome in the clipping group compared to coiling group except for the aneurysms in the posterior circulation^3,4,11–13^. Even lower rate of complete aneurysm occlusion and higher retreatment rate was demonstrated in the coiling group supporting the beneficial factors of clipping. Of note, most of the published studies dealt with saccular aneurysms; however there is paucity of literatures regarding microsurgical treatment of WNA, in particular. One recent meta-analysis including 2794 ruptured and unruptured wide neck bifurcation aneurysms reported 43.8% adequate occlusion rate in endovascular arm compared to 69.7% in microsurgical arm, while the safety events were comparable, 21% and 24.3%, respectively^14^. Furthermore, Mascitelli et al. performed posthoc analysis of WNA based on BRAT data and demonstrated significant higher complete occlusion rate in the clipping group with 84.3% compared to coiling group with 51.2%^11^. Over the 3- to 10 year’s follow-up, the occlusion grade dropped continuously in the coiling group whereas the outcome was stable with over 80% in the clipping group. Interestingly, the outcome did not differ at any time whereas the recanalization and retreatment rate was significantly higher in the coiling group^3,4,11^. This particular analysis was limited first to ruptured aneurysm, second wider range of inclusion criteria, neck width ≥ 4 mm or neck-to-dome ratio < 2, and last, to different endovascular treatment options. Therefore we focused the WNA definition to neck width ≥ 4 mm to build more homogenous cohort and widened our analysis to unruptured WNA besides ruptured WNA.

In general, WNA are difficult to treat due to wide-neck configuration, which is one of the prognostic factors for successful endovascular treatment^15^. Indeed the endovascular field on WNA was limited in the past; however through the development of new techniques like flow diverter, stent-assisted or ballon-assisted coiling, and recently novel technique with intrasaccular occluding device (WEB device), endovascular treatment became more effective and successful with stable results in the mid-term follow-up; although the long term data (> 10 years) are still missing^8,9^. To date, there have been 2 prospective multicenter trials, WEBCAST and the French Observatory trial, which reported complete occlusions rate of 56–52% and adequate occlusion rate of 86 to 79%, respectively^10^. Regarding complete occlusion rate, microsurgical treatment seems to be more effective compared to WEB-device, however, the question arises if it is necessary to achieve a complete occlusion of aneurysm^5^. In addition, a direct comparison is hard to perform since the main limitation would be the definition of an adequate occlusion and periprocedural complication. Besides recently, endovascular techniques like T-stenting assisted coiling has been introduced which show a comparable high complete occlusion grade of 73–83% in WNA with a stable result in the follow-up analysis and low complication rate^17,18^. Still one should notice that in contrast to microsurgical treatment, the retreatment rate of all alternative endovascular treatment is still higher, which is associated with a higher morbidity and mortality during long-term FU^16,19^. In our study, the complication rate of rWNA and uWNA was 15% and 8%, respectively. It seems a little high compared to general endovascular treatment (except WEB device), however, it is a matter of definition^19^. We included radiological features like epidural hematoma or brain insult, which was not clinically apparent except in the routine postoperative scan. Moreover, intraoperative rupture of aneurysm is questionable to calculate as complication since this happening is a normal state in an open surgery. In summary, the open-end question: clip or “coil” in WNA has still not been answered and a meta-analysis comparing endovascular and surgical treatment on WNA is warranted. Therefore our study might be an important addition to the literature in future for further comparison studies regarding outcome of WNA in both, ruptured and unruptured situation.

In our study, we tried to characterize the complexity of aneurysm configuration by comparing maximum- and perpendicular method. This method showed to be an independent predictor for microsurgical remnant besides early hydrocephalus and number of clips. Therefore this may serve as one of the preoperative measurement method to get a first insight of potential difficulty and alternative planning of operative approach. All in all, microsurgical treatment was effective and stable for ruptured as well as for unruptured WNA, thus, with respect to patient age and wishes, health status and local experience, we recommend more often microsurgical treatment in WNAs after interdisciplinary consensus.

### Limitations and generalizability

One limitation of this study is the limited follow-up of microsurgically treated patients. The reason for that was the absence of regular angiographic follow-up due to the known low number of recanalization after clipping^20,21^. Therefore, this bias may underestimate the recanalization rate, however, the radiographic follow-up was selected for high-risked patients for recurrence, which may result in rather overestimation. Moreover, Tsutsumi et al. have previously shown even lower risk of aneurysm growth of clipped aneurysm compared to de-novo aneurysm in mean follow up of 9 years indicating the stability of clipping in long-term follow-up^22^. Secondly, due to monocentric and retrospective design of our study, a bias of retrospective nature could have occurred, although we think that this would be small in regard of prospectively collected database. At last, there was no direct comparison of microsurgical treatment to endovascular treatment in our study wherefore no conclusion regarding superiority of each modality can be made. On the other hand, we think that due to the diverse experience of endovascular treatment between clinicians, those two arms would be better comparable within the scope of meta analysis.

## Conclusions

This study is one of the few studies reporting microsurgical outcome in rWNA and uWNA. Microsurgical clipping was effective for treatment of WNA with low complication rate. Moreover, remnants were mostly stable after clipping; the risk of growth was marginal even lower than the risk of de-novo rate resulting in hardly retreatment necessity. Still, further comparing studies regarding clip or newly developed endovascular alternatives are needed for a general consensus.

## Material and methods

**Figure 2:**
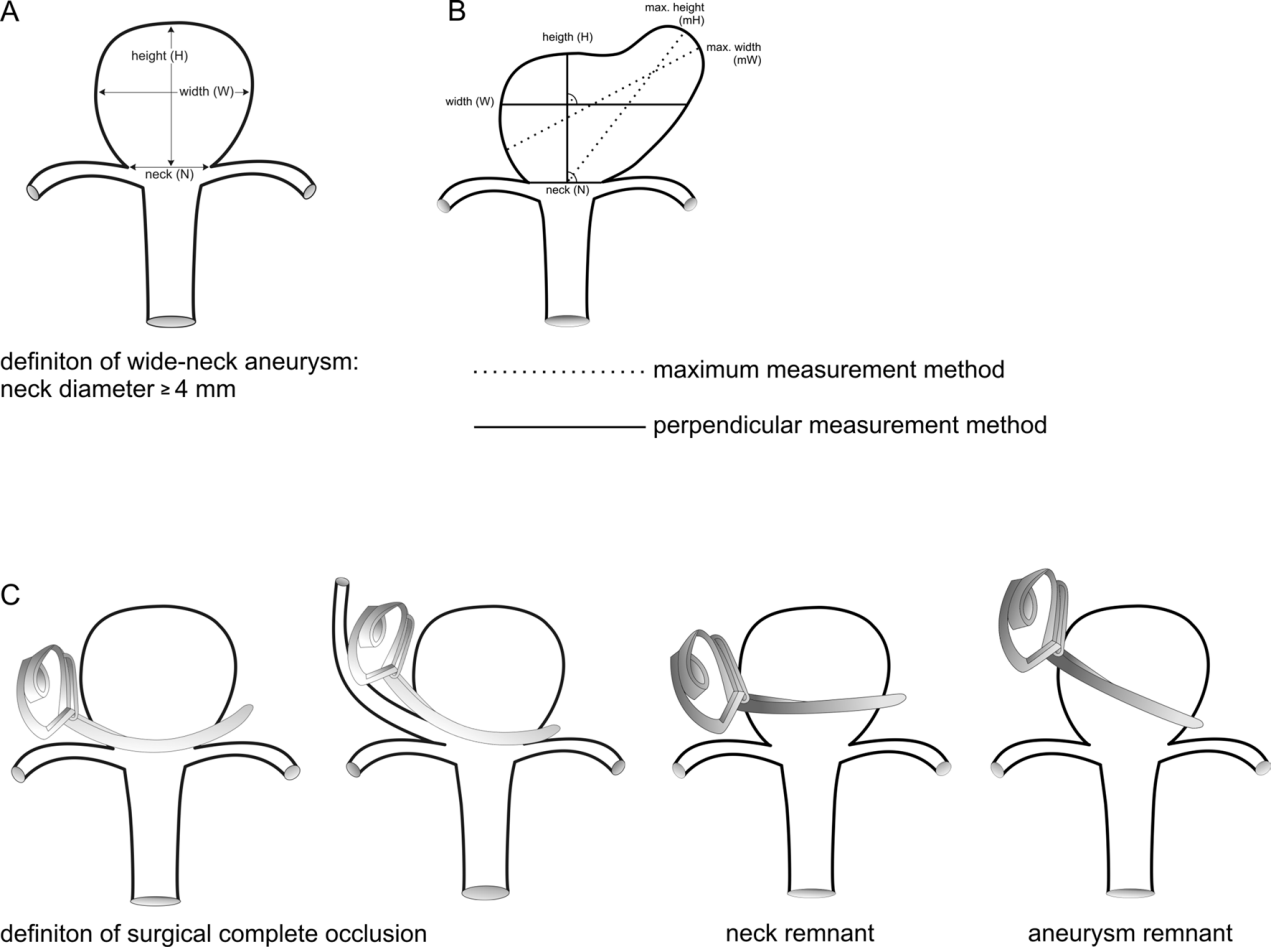
(**A**) Definition of wide neck aneurysm. (**B**) Differentiation between saccular perpendicular- (simple) and non-saccular non-perpendicular (complex) aneurysm. If width or height deviation was ≥ 2 mm, it was defined as a complex aneurysm. (**C**) Definition of Raymond-Roy occluding grade: complete, neck- and aneurysmal remnant.

This study received the authorization of the local ethical standards committee (IRB 19-245). In accordance with institutional rules, informed consent of anonymized patient was not required for this study. From a prospective collected database, a retrospective analysis was conducted with patients microsurgically treated on a WNA, either ruptured (rWNA) or unruptured (uWNA), from 2007 to 2017 in author´s institute. Inclusion criteria was the diagnosis of WNA, which was defined as maximum neck width $\ge$ 4 mm measured by the preoperative 3-dimensional 4-vessel digital subtraction angiography (DSA) oblique “working view” (Fig. 2A). Hendricks et al. summarized different definition of WNA in a systematic review reporting neck-to-dome ratio for further frequent used criteria. However, there were variable threshold defined for neck-to-dome ratio depending on the literatures, wherefore this parameter was not counted as definition criteria for WNA in our study.

All patients underwent preoperative DSA. Neck, dome and height size were measured in order to analyze WNA configuration in detail using DSA. In so doing, two different methods were applied to differentiate saccular (simple) and non-saccular (complex) aneurysm: 1. Maximum- 2. Perpendicular method (Fig. 2B). For the maximum-method, first the maximum neck diameter was measured and the maximal diameter regardless of perpendicularity of dome and height were calculated. For the perpendicular method, the neck size was the same as abovementioned; however, other parameters were calculated by drawing perpendicular line from neck to the height and additional dome line perpendicular to the height line. In case of less than 2 mm deviation of either neck or dome size, it was considered as saccular perpendicular (simple) aneurysm, otherwise the aneurysms were classified as non-saccular non-perpendicular (complex) aneurysm.

Microsurgical treatments were typically performed with or without rapid ventricular pacing depending on the size of aneurysm^6^. Afterwards, patients with rWNA underwent standard treatment according to SAH-guideline and patients with uWNA were followed up for a week. Besides the intraoperative ICG-angiography, microsurgical outcome was determined by a postoperative DSA 7 days after microsurgical treatment. Clinical follow-up was set at 6 months after treatment and depending on the microsurgical outcome, MRA or DSA were additionally recommended. Those data were used to analyze the mid-term microsurgical outcome.

The following information of each patient were completed: patient´s demographics including age, sex, smoker, family history of SAH, aneurysm information (location, size, neck/dome/height size), preoperative World Federation of Neurological Scale (WFNS), occurrence of hydrocephalus and cerebral vasospasm (CVS), delayed cerebral ischemia (DCI) and modified Ranking Scale (mRS) at discharge and 6 months’ follow-up (FU)^7^. WFNS 1–3 was considered as favorable neurological status at admission and favorable outcome was defined as mRS 0–2, otherwise it was defined as an unfavorable outcome. To mention, ICA-pcomm aneurysm was counted as aneurysm of anterior circulation, if pcomm aneurysm was included in the subsection of ICA. For all volumetric measurement (volume of WA, aneurysm remnants, SAH associated intracerebral hemorrhage), the simplified ABC/2 formula was used.

In general, this study was build up in 2 subsections:

1. Microsurgical outcome of rWNA at short- and midterm follow-up.
2. Microsurgical outcome of uWNA at short- and midterm follow-up.

As primary aim of the study, microsurgical outcome was evaluated on Raymond Roy occluding grade: complete occlusion (class I), neck remnant (class II) and aneurysm remnant (class III). We particularly used this classification in order to facilitate comparative analysis concerning endovascular treatment on WNA in the future. The modified Raymond Roy occluding grade was not used, because it was not applicable for surgical analysis. Complete occlusion was defined either as no more detectable aneurysm after clipping or intended neck remnant for the preservation of one or more perforators. Neck or aneurysm remnants were analyzed without knowledge of clinical outcome (Fig. 2C). Outcome at follow-up was analyzed by performing CT-angiography or DSA with following parameters: no remnant, remnant stable, remnant growth or de novo aneurysm. The secondary aim of the study was to determine predictors indicating postoperative aneurysm remnant.

### Statistical analysis

IBM SPSS Statistics (Version 22, IBM Corp.) was used for data analysis. Continuous variables were described as mean and standard deviation. Categorical variables were shown as a numerical order and percentage of the analyzed population. Univariate analyses as well as multivariate logistic regression analysis were performed to determine predictors for aneurysm remnant. Only parameters with p-value ≤ 0.05 were included in the regression analysis. For parametric variables, t-test was used and for non-parametric variables, Fisher´s exact test or chi-squared test were used as appropriate. To assess the impact of those variables, odds ratio with 95% confidence intervals were calculated. Statistical significance was defined as p-value ≤ 0.05 and all tests were 2-tailed.

### Ethical approval and informed consent

This study was approved by the local ethics committee of the Goethe University Hospital Frankfurt am Main, the methods were carried out in accordance with the relevant guidelines and regulations. For this retrospective study, informed consent was waived by local ethic committee of the Goethe University Hospital Frankfurt.

## Supplementary Information

Supplementary Information 1.

Supplementary Information 2.

Supplementary Information 3.

## Data Availability

The authors confirm that the data supporting the findings of this study are available within the article and its supplementary materials.
