# Supplementary material for: Short- and midterm outcome of ruptured and unruptured intracerebral wide-necked aneurysms with microsurgical treatment
Source: Sci Rep. 2021 Mar 2;11:4982. doi: 10.1038/s41598-021-84339-x (PMC7925666; doi:10.1038/s41598-021-84339-x)
Supplement: Supplementary file 1 — Supplementary Information 1. [file 41598_2021_84339_MOESM1_ESM.docx]

| **Supplementary Table e-1. Predictors for remnant after microsurgical treatment of ruptured wide-neck aneurysm** | | | | | | |
| --- | --- | --- | --- | --- | --- | --- |
| **Variable** | **No remnant** | **Remnant** | **Univariate analysis**  **p-value OR (95% Cl)** | | **Multivariate analysis**  **p-value OR (95% Cl)** | |
| No. of patients | 102 | 37 |  |  |  |  |
| Median age, yrs (range) | 52 (26-83) | 52 (2-64) | 1.0 |  |  |  |
| Sex |  |  |  |  |  |  |
| Male | 34 (33.3%) | 12 (32.4%) | 1.0 |  |  |  |
| Female | 68 (66.7%) | 25 (67.6%) | 1.0 |  |  |  |
| Smoker | 44 (43.1%) | 15 (40.5%) | 0.8 |  |  |  |
| WFNS 4-5 at admission | 44 (43.2%) | 25 (67.6%) | 0.01 | 2.7 (1.2-6.1) | 0.14 |  |
| WFNS |  |  |  |  |  |  |
| 1 | 33 (32.4%) | 9 (24.3%) | 0.4 |  |  |  |
| 2 | 14 (13.7%) | 1 (2.7%) | 0.1 |  |  |  |
| 3 | 11 (10.8%) | 2 (5.4%) | 0.5 |  |  |  |
| 4 | 7 (6.9%) | 3 (8.1%) | 1.0 |  |  |  |
| 5 | 37 (36.3%) | 22 (59.5%) | 0.03 | 2.5 (1.2-5.0) | 0.14 |  |
| Modified CT-Fisher score |  |  |  |  |  |  |
| 1 | 5 (4.9%) | 2 (5.4%) | 1.0 |  |  |  |
| 2 | 9 (8.8%) | 3 (8.1%) | 1.0 |  |  |  |
| 3 | 56 (54.9%) | 17 (46.0%) | 0.4 |  |  |  |
| 4 | 32 (31.4%) | 15 (40.5%) | 0.4 |  |  |  |
| ICH | 32 (31.4%) | 15 (40.5%) | 0.4 |  |  |  |
| <50ml | 20 (19.6%) | 10 (27%) | 0.4 |  |  |  |
| >50ml | 12 (11.8%) | 5 (13.5%) | 1.0 |  |  |  |
| Hydrocephalus | 65 (63.7%) | 31 (83.8%) | 0.04 | 9.1 (3.5-23.8) | 0.02 | 3.2 (1.2-8.8) |
| Shunt implantation within 6 months | 25 (24.5%) | 9 (24.3%) | 1.0 |  |  |  |
| Location of aneurysm |  |  |  |  |  |  |
| A1 | 3 (2.9%) | 0 (0%) | 0.6 |  |  |  |
| AcommA | 30 (29.4%) | 8 (21.6%) | 0.4 |  |  |  |
| A2 | 1 (1%) | 1 (2.7%) | 1.0 |  |  |  |
| A3/4 | 1 (1%) | 0 (0%) | 1.0 |  |  |  |
| ACI | 22 (21.6%) | 6 (16.2%) | 0.6 |  |  |  |
| -opthalmisch | 4 (3.9%) | 1 (2.7%) | 1.0 |  |  |  |
| -pcomm | 13 (12.7%) | 4 (10.8%) | 1.0 |  |  |  |
| -T | 5 (4.9%) | 1 (2.7%) | 0.7 |  |  |  |
| M1 | 1 (1%) | 2 (5.4%) | 0.2 |  |  |  |
| MCA-bifurcation | 35 (34.3%) | 15 (40.6%) | 0.6 |  |  |  |
| M2 | 4 (3.9%) | 1 (2.7%) | 1.0 |  |  |  |
| M3/4 | 1 (1%) | 1 (2.7%) | 1.0 |  |  |  |
| PCA | 2 (2%) | 0 (0%) | 1.0 |  |  |  |
| PICA | 2 (2%) | 1 (2.7%) | 1.0 |  |  |  |
| VA | 0 (0%) | 2 (5.4%) | 0.07 | NA | 0.42 |  |
| Aneurysm size, mm±SD | 8.7±4.2 | 9.6±4.2 | 0.3 |  |  |  |
| ≥10mm | 36 (35.3%) | 15 (40.5%) | 0.7 |  |  |  |
| ≥20mm | 2 (2%) | 2 (5.4%) | 0.6 |  |  |  |
| Neck size, mm±SD | 4.9±1.8 | 5.4±2.3 | 0.2 |  |  |  |
| Non-saccular nonperpendicular form of aneurysm | 74 (72.5%) | 20 (54.1%) | 0.04 | 2.5 (1.0-5.0) | 0.06 | 2.5 (1.0-5.0) |
| Dome to neck ratio |  |  |  |  |  |  |
| <1 | 16 (15.7%) | 9 (24.3%) | 0.3 |  |  |  |
| 1-1.9 | 57 (55.9%) | 16 (43.3%) | 0.2 |  |  |  |
| 2-2.9 | 25 (23.4%) | 10 (27%) | 0.8 |  |  |  |
| ≥3 | 4 (3.9%) | 2 (5.4%) | 1.0 |  |  |  |
|  |  |  |  |  |  |  |

ACA, anterior cerebral artery; AcomA, anterior communicans antery; ICA, internal carotid artery; MCA, media cerebral artery; PCA, posterior cerebral artery; PICA, posterior inferior cerebellar artery; VA, vertebral artery; CT, computer tomography.

NA: not applicable; Nagelkerke R^2^=0.20
