# Supplementary material for: Short- and midterm outcome of ruptured and unruptured intracerebral wide-necked aneurysms with microsurgical treatment
Source: Sci Rep. 2021 Mar 2;11:4982. doi: 10.1038/s41598-021-84339-x (PMC7925666; doi:10.1038/s41598-021-84339-x)
Supplement: Supplementary file 2 — Supplementary Information 2. [file 41598_2021_84339_MOESM2_ESM.docx]

| **Supplementary Table e-2.Predictors for remnant after microsurgical treatment of unruptured wide-neck aneurysm** | | | |
| --- | --- | --- | --- |
| **Variable** | **no remnant** | **remnant** | **p-value** |
| No. | 112 | 36 |  |
| Location of aneurysm |  |  |  |
| A1 | 2 (1.8%) | 1 (2.8%) | 1.0 |
| AcommA | 16 (14.3%) | 9 (25%) | 0.2 |
| A3/4 | 3 (2.7%) | 3 (8.3%) | 0.2 |
| ICA | 25 (22.3%) | 4 (11.1%) | 0.2 |
| -ophthalmisch | 13 (11.6%) | 2 (5.6%) | 0.4 |
| -pcomm | 6 (5.4%) | 2 (5.6%) | 1.0 |
| -bifurcation | 5 (4.5%) | 0 (0%) | 0.3 |
| M1 | 5 (4.5%) | 1 (2.8%) | 1.0 |
| MCA-bifurcation | 56 (50%) | 17 (47.2%) | 0.7 |
| M2 | 4 (3.6%) | 1 (2.8%) | 1.0 |
| PICA | 1 (0.9%) | 0 (0%) | 1.0 |
| VA | 1 (0.9%) | 0 (0%) | 1.0 |
| Aneurysm size, mm±SD | 9±5 | 10.6±5.4 | 0.1 |
| ≥10mm | 34 (30.4%) | 16 (16.7%) | 0.2 |
| ≥20mm | 5 (4.5%) | 3 (8.3%) | 0.4 |
| Neck size, mm±SD | 5±1.8 | 5.9±4.1 | 0.06 |
| Perpendicular form of aneurysm (2mm cutoff) | 59 (51.8%) | 13 (36.1%) | 0.08* |
| Dome to neck ratio |  |  |  |
| <1 | 20 (17.9%) | 6 (16.7%) | 1.0 |
| 1-1.9 | 67 (59.8%) | 22 (61.1%) | 0.8 |
| 2-2.9 | 20 (17.9%) | 7 (19.4%) | 1.0 |
| ≥3 | 5 (4.5%) | 1 (2.8%) | 1.0 |
|  |  |  |  |
| Median age, yrs (range) | 51 (16-73) | 52 (33-71) | 1.0 |
| Sex |  |  |  |
| Male | 25 (22.3%) | 11 (30.6%) | 0.3 |
| Female | 87 (77.7%) | 25 (69.4%) | 0.3 |
| Familiar history of aneurysm | 9 (8%) | 3 (8.3%) | 1.0 |
| Previous SAH | 14 (12.5%) | 4 (11.1%) | 1.0 |
| Smoker | 60 (53.6%) | 20 (55.6%) | 1.0 |
| *Chi-square test |  |  |  |
