# Supplementary material for: Short- and midterm outcome of ruptured and unruptured intracerebral wide-necked aneurysms with microsurgical treatment
Source: Sci Rep. 2021 Mar 2;11:4982. doi: 10.1038/s41598-021-84339-x (PMC7925666; doi:10.1038/s41598-021-84339-x)

Figure e-1. Short- and midterm outcome of ruptured and unruptured intracerebral wide-necked aneurysms with microsurgical treatment. Sae-Yeon Won MD; Volker Seifert MD, PhD, Daniel Dubinski MD, MSc; Sepide Kashefiolasl MD; Nazife Dinc MD; Markus Bruder MD; Juergen Konczalla MD,PhD.
A. Patient with an unruptured wide neck aneurysm of media cerebral artery. **B.** Postoperative angiography with illustration of a filiform aneurysmal remnant of a size of 6.5mm. **C.** Angiography after 1 year follow-up showing spontaneous disappearance of the aneurysmal remnant.

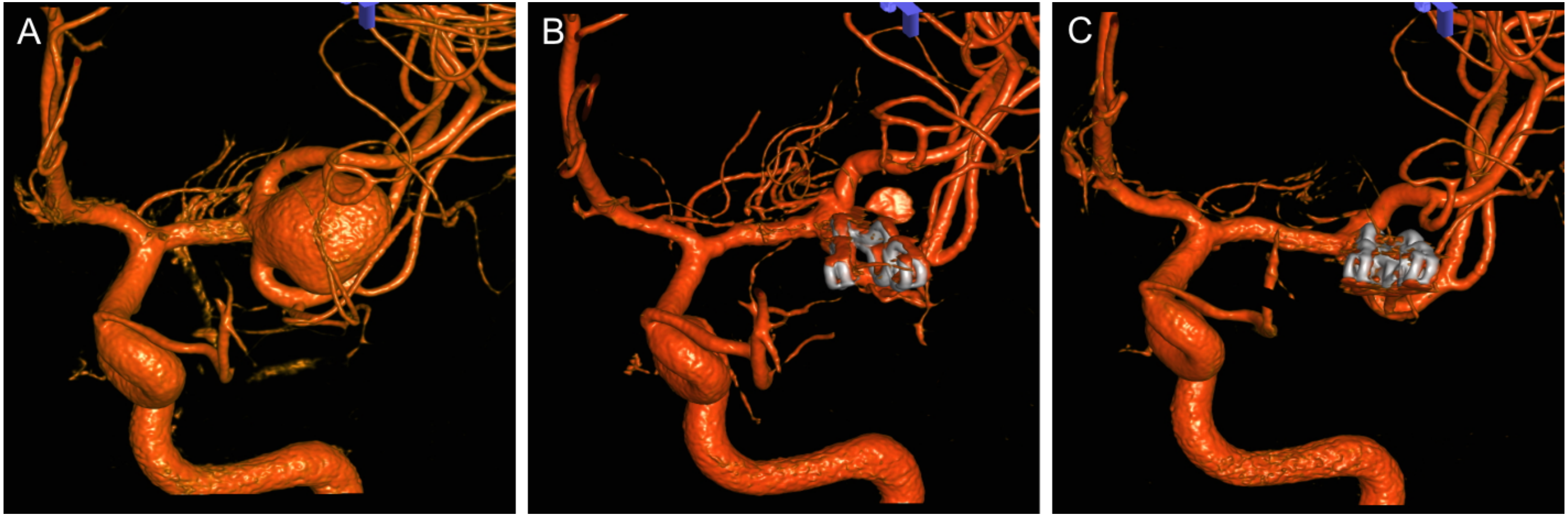

Supplement: Supplementary file 3 — Supplementary Information 3. [file 41598_2021_84339_MOESM3_ESM.pdf]
